# Co -delivery of Sulforaphane and Curcumin with PEGylated Iron Oxide-Gold Core Shell Nanoparticles for Delivery to Breast Cancer Cell Line

**Published:** 2018

**Authors:** Hossein Danafar, Ali Sharaﬁ, Shaghayegh kheiri, Hamidreza Kheiri Manjili

**Affiliations:** a *Cancer Gene Therapy Research Center, Zanjan University of Medical Sciences, Zanjan, Iran. *; b *Zanjan Pharmaceutical Biotechnology Research Center, Zanjan University of Medical Sciences, Zanjan, Iran.*; c *Department of* *Agricultural Manegement,Islamic Azad University, Abhar Branch, Zanjan, Iran.*

**Keywords:** Apoptosis, Breast cancer, Cancer therapy, Curcumin, PEGylated gold-coated Fe3O4 nanoparticles, Sulforaphane

## Abstract

Co-delivery approach has been recommended to reduce the amount of each drug and to achieve the synergistic effect for cancer treatment. Curcumin (CUR) and sulforaphane (SF) have antitumor effects, but their application is limited because of their low water solubility and poor oral bioavailability. To improve the bioavailability and solubility of SF and CUR, we performed an innovative co-delivery of them with PEGylated gold coated Fe_3_O_4_ magnetic nanoparticles (PEGylated Fe_3_O_4_@Au NPs) to endorse SF and CUR maintenance as an effective and promising antitumor drugs. The structure of the synthesized nanocarriers evaluated by X-ray diffraction, transmission electron microscopy, scanning electron microscopy, vibrating sample magnetometer, dynamic light scattering and Fourier transform infrared spectroscopy. The results revealed that the zeta potential of CUR and SF-loaded NPs were about -15.4 mV and the average sizes were 80.57 nm. They were monodispersed (polydispersity index = 0.161 ± 0.016) in water with high drug-loading capacity and stability. CUR and SF were encapsulated into NPs with loading capacity of 17.32 ± 0.023% and 16.74 ± 0.015% and the entrapment efficiency of 83.72 ± 0.14% and 81.20 ± 0.18% respectively. The *in-vitro* study of SF and CUR loaded PEGylated Fe_3_O_4_@Au NPs on human breast adenocarcinoma cell line (MCF-7) confirmed that cytotoxicity of SF and CUR can enhance when they are loaded on PEGylated Fe_3_O_4_@Au NPs in comparison to free SF and CUR. The results of real-time PCR and flow cytometry shown that this combination can increase therapeutic effects of SF and CUR by apoptosis and necrosis induction as well as inhibiting of migration in MCF-7 cell line.

## Introduction

Mostly, synergistic mixture of two or more medications is a favorable method to overcome unwanted toxicity and other side effects which can restrict the use of many potential drugs (1-4). Simultaneous delivery of different drugs with different physiochemical properties to the same tumor cells after a single injection are known as co delivery systems (5, 6). Recently, many multifunctional drug delivery systems including nanoparticles (NPs), liposomes and inorganic nanoparticles have been designed for co-delivery of different therapeutic agents (7). Over the past 30 years, many efforts have been performed to synthesize and modify nanoparticles for biomedical applications (8). Recently, a large number of studies have been devoted to the magnetic nanoparticles (MNPs) due to their potential abilities. MNPs have been used for therapeutic targets such as cancer treatment (9, 10), diagnosis, magnetic resonance imaging (MRI), contrast agents, magnetic cell separation and immunoassays. Targeted drug delivery using MNPs is an attractive method to improve the performance of cancer therapy (11). The ideal drug delivery system (DDS) presents a safe and non-toxic formulation of traditional drug which can selectively kill cancer cells without damaging normal cells. Valuable libraries of DDSs are feasible using different nano-materials with different features such as proper size, shape, surface charge and stability for *in-vitro* and *in-vivo* uses. Among them, magnetic iron oxide (Fe_3_O_4_) is very important because of its fine size and biocompatibility (12-14). Other advantages of MNPs are the super paramagnetic property, non-poisonousness, lifelessness and simple detection in the human body. MNPs having a large surface area to volume ratio and low surface charge at neutral pH, dispersions of these particles typically have low stability with the MNPs tending to aggregate when dispersed in solvents. Such aggregation can be reduced with appropriate surface chemistry (15). However, the surfaces of most magnetic materials are not highly compatible with well-defined surface chemistry such as the alkanethiol system. Gold coating of the MNPs addresses all the above mentioned challenges including conductivity, optical properties biocompatibility,(16, 17) bio affinity through functionalization of amine/thiol terminal groups (18),  and chemical stability by protecting the magnetic core from aggregation, oxidation and corrosion (19).

Natural products have been used for the treatment of various diseases (20, 21). sulforaphane (SF) is a chemo-preventive agent which can reduce (22), postpone or reverse the process of carcinogenesis and is known as a favorable and powerful anti-carcinogen in different types of cancers (23, 24). SF is an isothiocyanate compound found in broccoli, cauliflower, kale and other cruciferous vegetables, which chemically named 1-isothiocyanato-4-(methylsulfinyl) butane. Because of the SF instability and its sensitivity to oxygen, heat and alkaline conditions, it is difficult to manufacture and distribute it in pharmaceutical industries (25-27). It has been reported that the cancer chemo-protection of SF can induce through a range of mechanisms such as apoptosis induction, cell cycle arrest, inhibiting angiogenesis and cancer metastasis (28), anti-inflammatory activity and inhibiting cytochrome P450 activity following activation of deoxidification enzymes. SF potently prevents angiogenesis of tumors and metastasis by reducing formation of micro-capillary and inhibiting cell migration (29-31). Due to high surface area, tunable stability and low inherent toxicity, gold based nano-systems have developed as promising frameworks for drug delivery vehicles. There are a large number of efforts to improve the particle surface for improving properties such as efficiency, bioavailability and non-immunogenicity. Also, longer circulation time and more tumor accumulation are achieved by PEG-stabilized Gold Nanorods (Au NRs).

Curcumin (CUR), a chemical constituent of turmeric, has been used in the treatment of inflammatory disorders and cancers for many years (32). There is some data signifying that CUR is a principle chemo-sensitizer for chemotherapy and it can protect patients from the side effects of treatments (33). CUR can reduce tumor growth by various mechanisms including antitumor angiogenesis, suppression of proliferation (34, 35), induction of apoptosis (36, 37) and prevention of metastasis (38, 39). However, the clinical applications of CUR remain incomplete because of its short biological half-life, poor solubility resulting in poor absorption and low bioavailability *via* the oral route (40-42). Development of an intravenous preparation is a promising method to resolving these issues in the CUR application. Nanotechnology has the potential to conquest of the poor water solubility of lipophilic drugs. Encapsulation of hydrophobic drugs into nanoparticles is a confident progress to formulate the drug intravenously injectable (43). 

Therefore, PEGylated Au NRs are believed to be more promising. They have attracted increasing attentions compared to other types of Au NPs (44-46). Based on our knowledge, there are no reports on the applying of nano-vehicles for co-delivery of SF and CUR to cancerous cells. Therefore, the SF and CUR instability and its sensitivity to condition can be overcome by designing a desirable delivery system. Consequently, based on the value SF and CUR medicinal properties and in continuation of our interest in the field of nano-materials, we present an efficient nano-DDS to enhance the absorption and therapeutic level of SF and CUR in the breast cancer cell line (MCF-7). We will suggest that SF and CUR loaded PEGylated Fe_3_O_4_@Au NPs are the good candidate for delivery of SF and CUR in the breast cancer cell line.

## Experimental

*Materials*

MTT (Aldrich, St. Louis, USA, CAS. 57360-69-7),  (E,E)-1,7-bis (4-Hydroxy-3-methoxyphenyl)-1,6-heptadiene-3,5-dione, Diferuloylmethane, Diferulylmethane, Curcumin (Merck, Darmstadt, Germany, Art No. 820354), 1-Isothiocyanato-4-(methylsulfinyl)-butane (DL-Sulforaphane) (Aldrich, St. Louis, USA, CAS. 4478-93-7),  The human breast adenocarcinoma cell line MCF-7 and mouse mammary tumor cell line 4T1  were purchased from the National Cell Bank of Iran (Pasteur Institute, Iran), No ethics statement was required from the institutional review board for the use of these cell lines. Dulbeccoʹs modified Eagleʹs medium (DMEM) (Gibco, Germany), L-glutamine, penicillin, streptomycin, Fetal Bovine Serum (FBS) (Gibco, Germany), Fe_3_O_4_ , Other chemicals and solvent were from chemical lab purity grades, purchased from Emertat chimi Co.

*Synthesis of PEGylated Fe3O4@Au NPs*

With some modifications, Fe_3_O_4 _and Fe_3_O_4_@Au NPs were synthesized according to the previous descriptions (47-49). Briefly, a 2:1 ratio of ferric and ferrous chloride (2 M) was dissolved in deionized water, and the pH reached to 10 by adding ammonia. This was followed by 1 h stirring at room temperature for 30 min at 80 °C, until the dark MNPs appeared in the solution. An external magnetic field was applied to separate Fe_3_O_4_ NPs from the solution (47). Then, a 2% solution of Fe_3_O_4_ NPs prepared in sodium citrate (10 mM) and after sonication was slowly heated to 70 °C. Afterwards, 1 mL of 0.1 M solution of HAuCl_4_.4H_2_O was dropped into the solution and stirred vigorously for approximately 30 min, allowing Au^3+^ to attach to the surface of the MNPs. Finally, a magnetic field was used again to purify the Fe_3_O_4_@Au from Au NPs. Further, 0.04 g of Fe_3_O_4_@Au dispersed in 5 mL

 of CHCl_3_ was added to 0.03 g thiolated polyethylene glycol (HS-PEG-OMe) in CHCl_3_. The mixture was stirred for 2 h in the dark at room temperature. The particles were collected by an external magnetic device or centrifugation and washed with CHCl_3_ and hexane (1:5 V/V). To obtain pure PEGylated Fe_3_O_4_@Au NPs, the resulting product was dialyzed for 24 h.

*Preparation of SF/CUR-loaded Fe3O4@Au NPs*

20 mL of Fe_3_O_4_@Au NPs with the final concentration of 0.5 mg/mL was sonicated with 5 mL stock solution of SF and CUR with an initial concentration of 0.5 mg/mL for 30 min and then stirred overnight at RT in dark. The samples were separated applying an external magnet device, and washed with dry ethanol for three times. 

The SF and CUR concentration in the liquid layer were measured using a standard SF/CUR concentration curve generated with an UV-Visible spectrophotometer at 235 and 420 nm respectably from a series of SF and CUR solutions with different concentrations. 

*Method Validation*

The method was validated for selectivity, linearity, accuracy, precision, recovery, detection limit and quantitation limit according to the principles of the FDA industry guidance. 

*Determination of loading efficiency*

To determine the loading efficiency of the drugs in the micelles, two parameters including the drug loading ratio and encapsulation efficacy were evaluated. Drug loading ratio (DL) was determined as:

$\mathrm{DL\%}=\frac{\mathrm{Wdrug in NPs}}{\mathrm{WNPs}}\times 100$

Eq. 1

Wdrug in NPs and WNPs show weight of the encapsulated drug and the total weight NPs, respectively. For determination of the drug loading ratio, 1 mg of the final freeze-dried nanodispersion NPs was dissolved in 1 mL of NaOH 1N, and the drug content SF and CUR was measured by UV-Vis (Thermo Fisher Scientific, USA, Madison, model GENESYS™ 10S) at wavelength of at 235 and 420 nm respectively.

Encapsulation efficacy (EE) was determined using the following equation:

$\mathrm{EE\%}=\frac{\left(\mathrm{W drug in NPs}\right)}{\mathrm{W initial drug}}\times 100$

Eq. 2

*Characterization of PEGylated Fe*_3_ *O*_4_ *@Au NPs*

For study of the sizes of NPs, the samples were observed by TEM at the voltage of 80 KV and morphological characteristics of images obtained by SEM. X-ray diffraction (XRD) data were measured by a Philips X’pert 1710 diffractometer using Cu Kα (α = 1.54056 Å) in the Bragg-Brentano geometry (Ө-2Ө) to determine the composition structure of NPs. Magnetic properties were evaluated by vibrating sample magnetometer. The FTIR spectra were taken in the region of 4000–400 cm^−1^. Aggregation levels of Fe_3_O_4_ compared with Fe_3_O_4_@Au were investigated under an inverted microscope (Olympus, IX81) and digital images were captured with a DP72 CCD camera.

*Determination of particle size*

The organized NPs size distribution was gave by DLS using a nano/zetasizer (Malvern).

*Drug release study*

The release of SF and CUR from PEGylated Fe_3_O_4_@Au NPs was obtained at 2-120 h and at pH 7.4 and 5.4. PEGylated Fe_3_O_4_@Au NPs (20 mg) dissolved in 5 mL of phosphate buffered saline buffer containing 0.5% (w/w) of Tween-80 and added into the dialysis bags at 37 °C with gentle shaking. 2 mL of samples were collected and replaced with 2 mL of same fresh buffer solution. During 5 days, drug releases were evaluated by a UV-visible spectrophotometer at 235 and 420 nm for SF and CUR respectively at different time intervals. As controls, the release of free CUR and free SF was studied in PBS. All the release studies were carried out in triplicate.

*MTT Assay*

*Cell lines, cell cultures and characterization experiments*

The cells were cultured in DMEM supplemented with 2 mM L-glutamine, penicillin (50 IU/mL), streptomycin (50 μg/mL) and 10% FBS (Gibco, Germany), incubated at 37 ºC in a humidified incubator with 5% CO_2_. Then they were detached using 0.25% trypsin and 0.53 mM EDTA in PBS. 

*Analysis of cytotoxicity*

The cytotoxicity of free CUR, SF and the drug-loaded PEGylated Fe_3_O_4_@Au nanoparticles on the MCF-7 and 4T1 cell lines were evaluated by the MTT method. Briefly, MCF-7 and 4T1 cells were plated at a density of 5×10^3^ cells per well in 100 μL of DMEM containing 10% FBS in 96-well plates and grown for 24 h. Cells were then exposed to a series of free CUR, SF or drug-loaded PEGylated Fe_3_O_4_@Au nanoparticles at different concentrations for 12, 24, 48 and 72 h. The viability of cells was measured using the MTT method. Results were the mean of five test runs. The combined indexes for the SF/CUR combination at different mass ratios were calculated as before described. The cell viability index was calculated according to the following Equations:

Eq.3 Cytotoxicity % = 1− (Mean absorbance of toxicant / Mean absorbance of negative control)

Eq. 4 Viability % = 100−Cytotoxicity %

*Apoptosis detection*

The apoptosis was identified by Annexin V–FITC and propidium iodide (PI) staining. MCF-7 cells were planted into six-well plates and exposed to a series of nanoparticles separately. Cells treated with normal saline were considered as the control group. The treated cells were collected, washed and then stained for 20 min. The ratio of apoptotic cells was evaluated by flow cytometry (BD, USA).

*Real-Time Polymerase Chain Reaction with SYBR Green I*

Total RNA from cells was extracted using an RNA isolation kit (Sigma Co.). cDNAs were synthesized using Qiagen Co. kit based on the manufacturer’s instructions. cDNAs were stored at -20 °C. Real-time PCR was carried out in a 20 µL reaction solution using sequences specific primers (Table 1) in optical grade 96-well plates.

Thermal cycling was performed on the ABI 7300 real-time PCR system. Threshold cycle (Ct) data were collected using ABI Prism 7300 sequence detection system version 1.2.3.

The relative gene expression was analyzed by the 2^- ΔΔCt^ method. The fold change in target gene cDNA relative to the GAPDH (Glyceraldehyde-3-Phosphate Dehydrogenase) internal control was calculated by:

Fold change = 2^- ΔΔCt^

ΔΔCt = sample (^Ct^ Target gene - ^Ct^ GAPDH) – Reference sample (^Ct^ Target gene -^Ct ^GAPDH)

*Evaluation of toxicity to the heart, spleen, liver and kidney*

For the experiment, 6 to 8 weeks old female inbred Balb/c mice were purchased from Pasteur institute of Iran (Karaj, Iran). Our laboratory animal facility is maintained under a 12-h light/dark cycle at a temperature of 20–24 C and a relative humidity of 20–30 %. Based on systematic studies for identification of dose-range as well as practical consideration of the synthetic capacity of Fe_3_O_4_@Au NPs, different concentrations of Fe_3_O_4_@Au NPs were administered to the mice intraperitoneally for 2 weeks. 14 days after drug treatment, the mice were killed by cervical vertebra dislocation. Heart, liver, spleen and kidney tissues were picked, ﬁxed in 10% (v/v) neutral buffered formalin, embedded in parafﬁn, sectioned at 4 μm thickness and stained with hematoxylin and eosin (50, 51) for histopathology analysis.

*Statistical analysis*

Statistical analysis was performed with one-way analysis of variance using SPSS software. *P*-values < 0.01 were considered to be statistically significant.

## Results and Discussion

*Preparation and Characterization of Nano-carrier*

**Figure 1:**
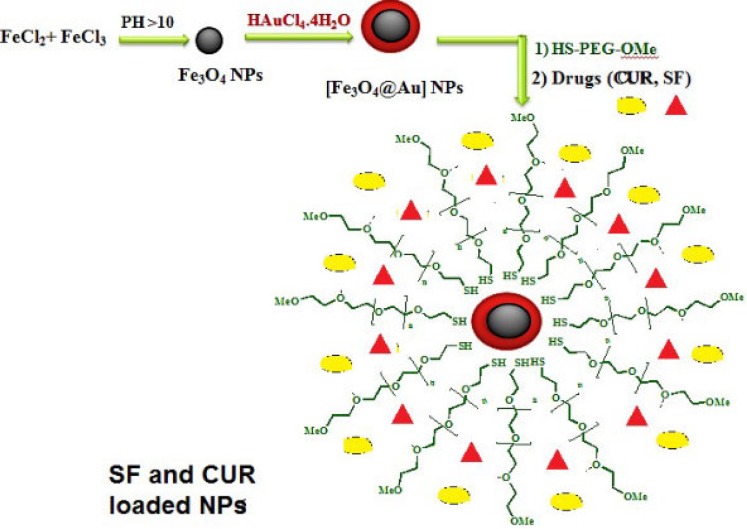
**Schematic representation of SF/CUR loaded PEGylated Fe**_3_ **O**_4_ **@Au NPs preparation.**

**Figure 2:**
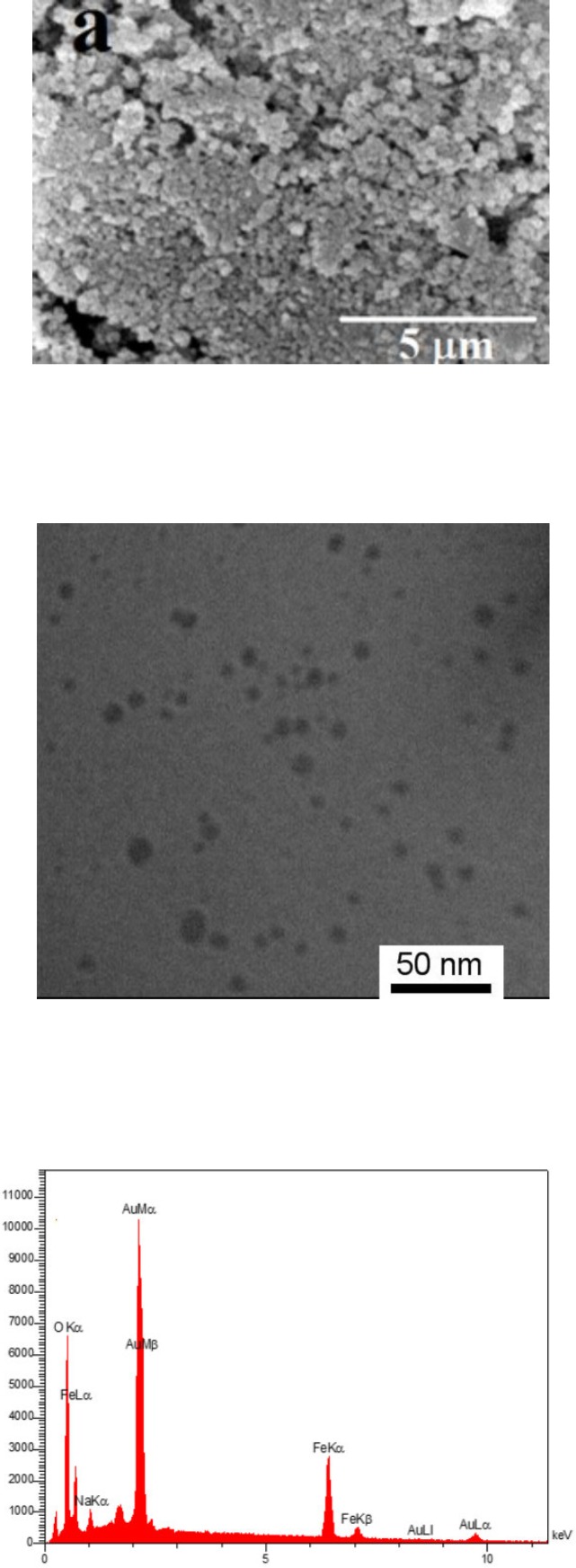
**a) SEM, (b and c) TEM, and EDX images of Fe**_3_ **O**_4_ **@Au NPs.**

**Figure 3:**
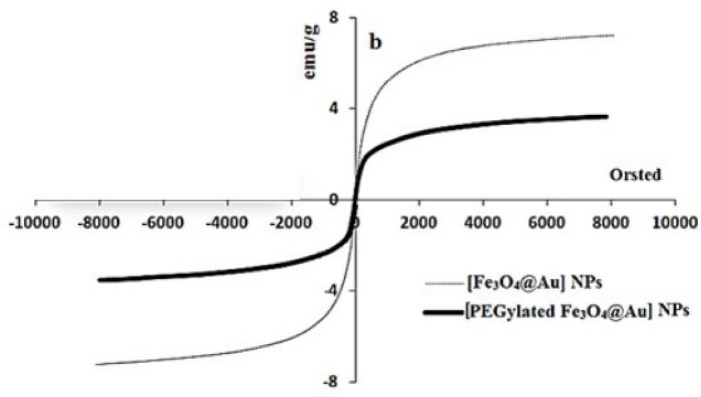
Magnetic hysteresis measurements of NPs using VSM

The gold coated Fe_3_O_4_ NPs including Fe_3_O_4_@Au, PEGylated Fe_3_O_4_@Au, and SF and CUR loaded PEGylated Fe_3_O_4_@Au NPs were prepared as shown in Figure 1. Fe_3_O_4_@Au NPs were synthesized *via* co-precipitation of Fe^2+^ and Fe^3+^ ions and followed by coating resulting Fe_3_O_4_ NPs with gold NPs. Then, the Fe_3_O_4_@Au NPs were modified by thiolated polyethylene glycol (HS-PEG-OMe) to fabricate PEGylated Fe_3_O_4_@Au NPs as a magnetic nano-carrier. SEM and TEM were used to recognize the morphology of the Fe_3_O_4_@Au NPs. As shown in the SEM (Figure 2a) and TEM (Figure 2b), the average size of synthesized encapsulated NPs is smaller than 50 nm after the PEGylation. To confirm the existence of iron oxide-gold core-shell NPs, the powder EDX experiment was utilized as a very sensitive and applicable technique. The three bands shown in the EDX pattern are clearly attributable to the corresponding reflections of Au are present at 2θ = 44.67, 51.72 and 76.71 (47). However, the bands characteristic of magnetic core no longer appears in the EDX of the final product (Figure 2c) (52). It is important that the core-shell material possess sufficient magnetic and super paramagnetic properties for practical applications (53, 54). Magnetic hysteresis measurements for the Fe_3_O_4_@Au and PEGylated Fe_3_O_4_@Au NPs were performed in an applied magnetic field at room temperature, with the field sweeping from −8000 to +8000 Oe. As shown in Figure 3, the M-H hysteresis loop for the samples was completely reversible representing that the NPs exhibit super paramagnetic characteristics. The lower the magnetic saturation of later NPs could be due to the influence of the PEG-functional group.

**Figure 4.**
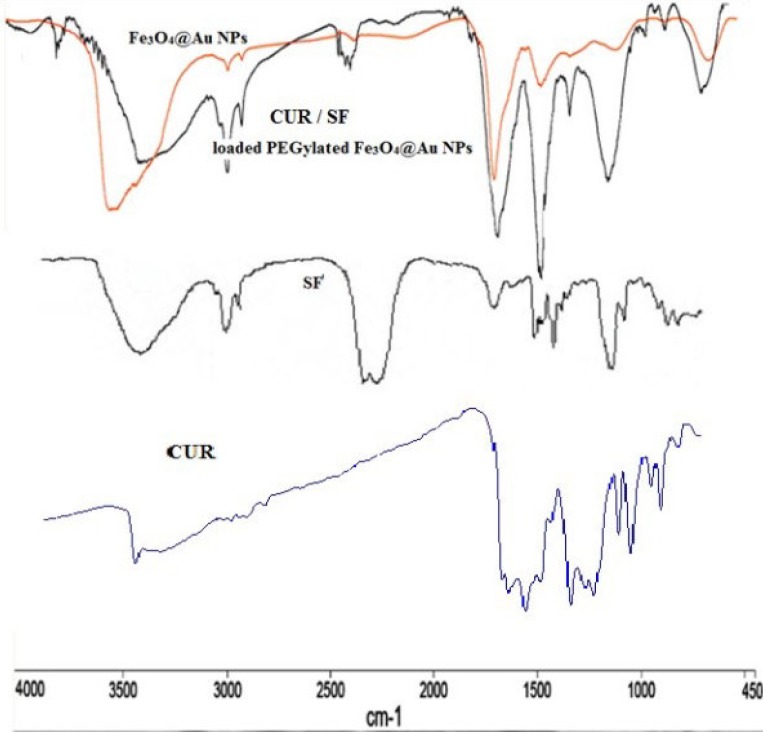
FTIR spectra of SF, CUR and NPs

**Figure 5:**
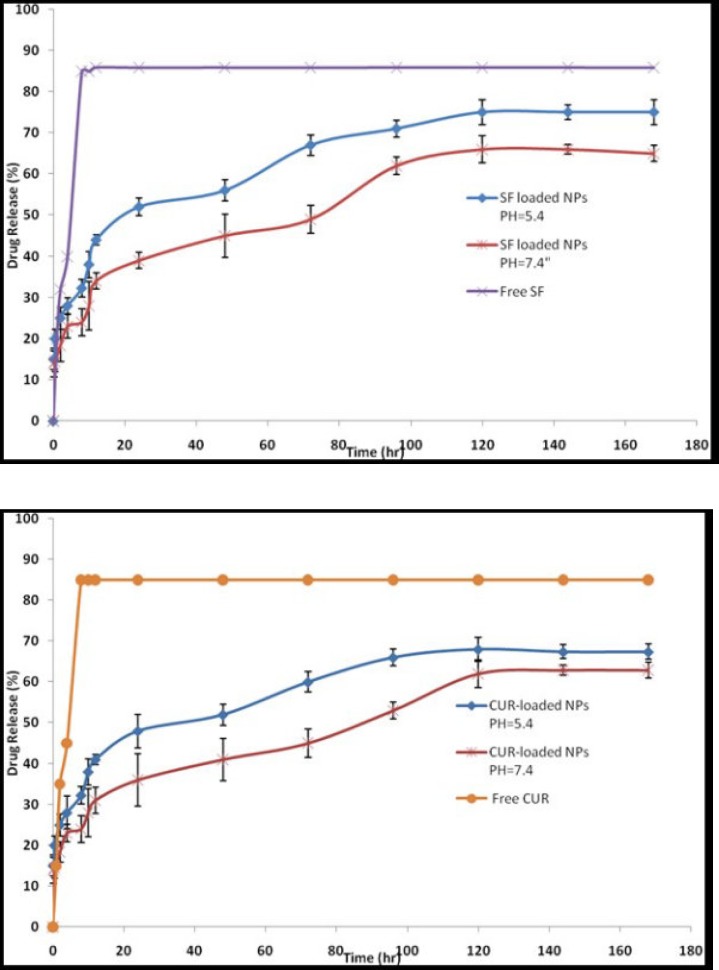
**The release profiles of SF and CUR from SF/CUR loaded PEGylated Fe**_3_ **O**_4_ **@Au NPs in different release media (a) pH = 7.4, (b) pH = 5.4**

The FTIR spectra of Fe_3_O_4_@Au and SF and CUR loaded PEGylated Fe_3_O_4_@Au NPs were performed and confirmed the successful loading of SF and CUR onto the PEGylated Fe_3_O_4_@Au NPs (Figure 4). In the FTIR spectrum of SF and CUR loaded PEGylated Fe_3_O_4_@Au NPs compared to Fe_3_O_4_@Au NPs, the appeared strong absorbance peaks at 2250 and 2280 cm^−1^ should be for the –N = C = S stretching vibration. Also, the absorption peaks at 1077, 1274, and 620 cm^−1^ are respectively assigned to the S = O (and C-O bond of PEG), C–N and C–S bonds. In addition, the absorption peak at1398 cm^−1 ^is noted for CH_3_. The size of nanoparticles was measured by dynamic light scattering technique. As shown in Figure. 5, the z-average and zeta potential of CUR / SF loaded NPs is 80.57 nm, with zeta potential -15.4 mv and their corresponding PDI is 0.161.

**Table 1:** Characteristics of the Primers Used in the Real-Time Polymerase Chain Reaction

**Gene**	**Sequence**	**Amplicon Size (bp)**	**Tm °C**
GAPDH (F)	ACTAACCCTGCGCTCCTG	87	59.03
GAPDH (R)	CCCAATACGACCAAATCAGA		59.04
Bcl-XL (F)	AACTGTGGTTCAGTGTGGGA	87	59.13
Bcl-XL (R)	CTAGCTTCCTCCAAGATGGC		59.54
BCL-2 (F)	CTTTACGTGGCCTGTTTCAA	88	58.68
BCL-2 (R)	CTGAAGGACAGCCATGAGAA		58.90
BAX(F)	AGCTGCAGAGGATGATTGC	153	59.43
BAX(R)	GTTGAAGTTGCCGTCAGAAA		58.82

**Table 2:** Within-run precision, between run precision, accuracy and recovery of CUR and SF.

**Nominal added** **concentration** **(mg/mL)**	**Accuracy±SD** **CUR**	**Accuracy±SD** **SF**	**Recovery±SD** **CUR**	**Recovery±SD** **SF**	**Within-run** **precision** **CUR**	**Within-run** **precision** **SF**	**Between-run** **precision** **CUR**	**Between-run** **precision** **SF**
0.1	96±1.03	95±1.4	98±0.21	93±0.36	3.2	4.5	5.8	1.9
0.5	94±1.2	93±0.65	96±0.87	96±1.1	5.6	9.8	7.6	4.1
1	97±0.32	98±0.2	92±1.12	98±0.98	7.6	9.0	6.9	6.7

**Figure 6:**
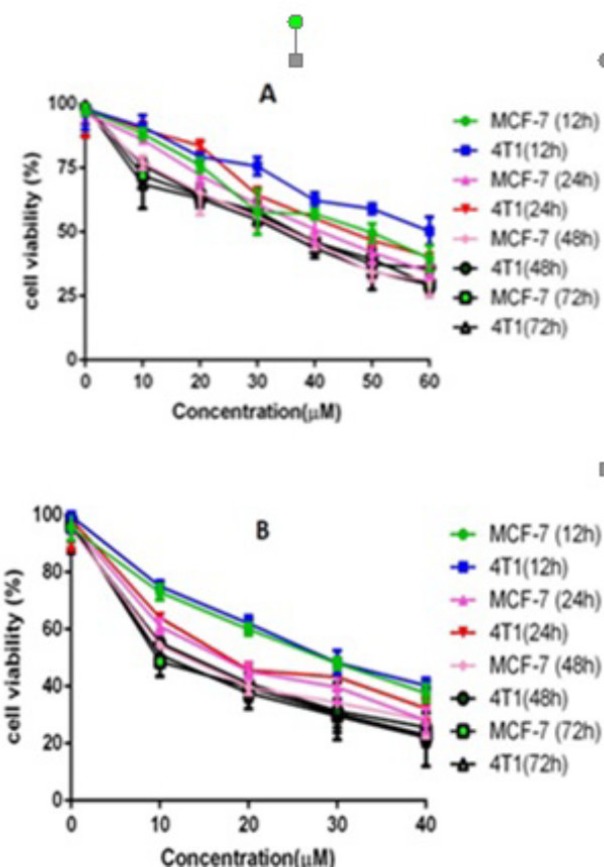
Anticancer abilities of free SF and CUR on MCF-7 and 4T1 Breast cancer cells *in-vitro*.

Notes: (A) CUR; (B) SF. Abbreviations: SF; sulforaphane, CUR; curcumin.

**Figure 7.**
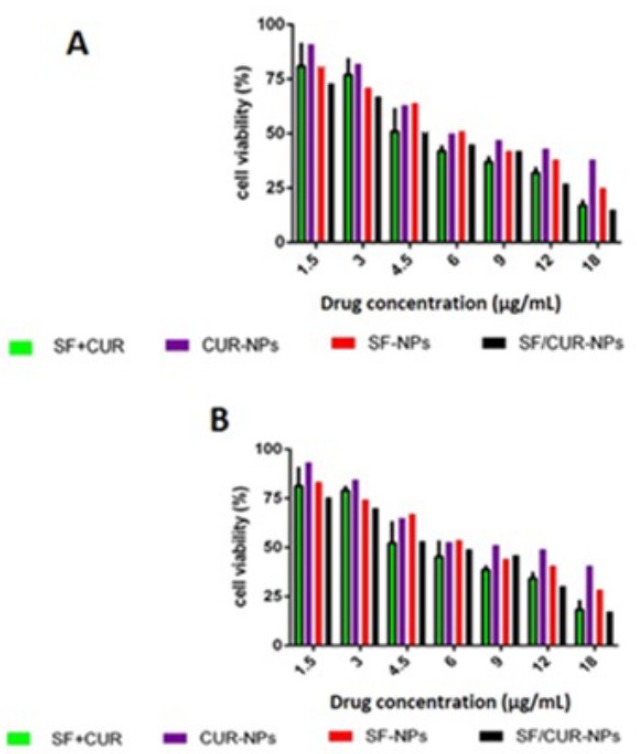
Anticancer abilities of different formulations containing SF and/or CUR against MCF-7 and 4T1 cells after treatment for 72 h. Notes: (A) MCF-7 cells; (B) 4T1cells.Abbreviations: SF; sulforaphanecur, CUR; curcumin

**Figure 8.**
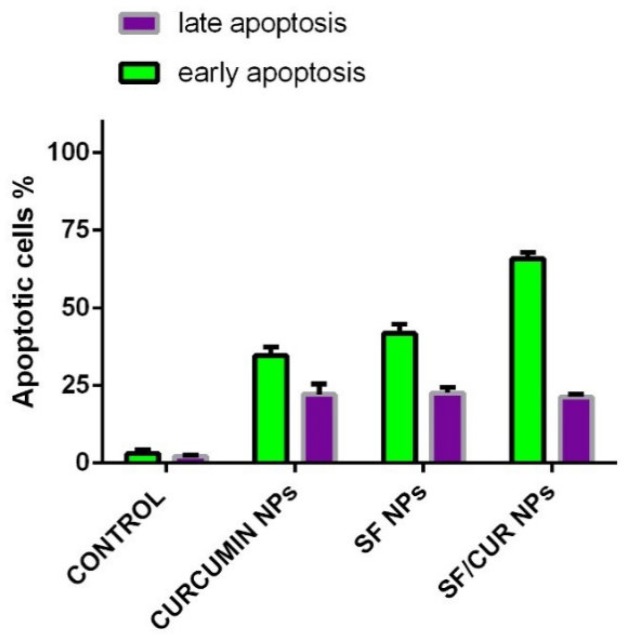
Flow Cytometry results illustrate the percentages of early and late apoptotic cells between SF-NPs, Fe_3_O_4_@Au NP, CUR-NPs and SF/CUR-loadedPEGylated Fe_3_O_4_@AuNPs

**Figure 9:**
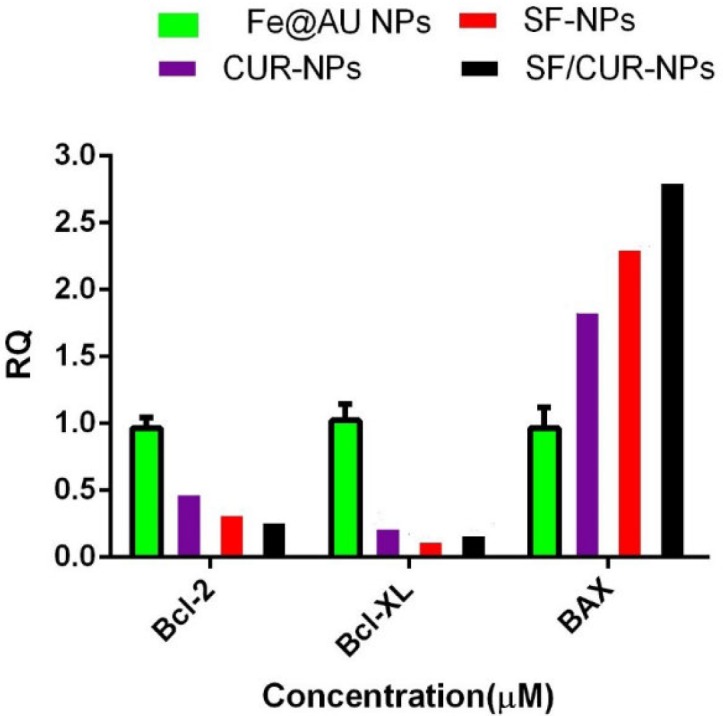
Statistical analysis of Real Time-PCR results by two-way ANOVA and Bonferroni posttest at the MCF-7 cells that were treated by SF/CUR-codelivery PEGylated Fe_3_O_4_@Au NPs nanoparticles, CUR-NPs, SF-NPs and Fe3O4@Au NPs as control. Mean ± SD. n=5. The symbols beside each group’s indicator present *P 0.1, **P 0.01,* **P 0.001 and****P 0.0001 significant difference against that control group

**Figure 10:**
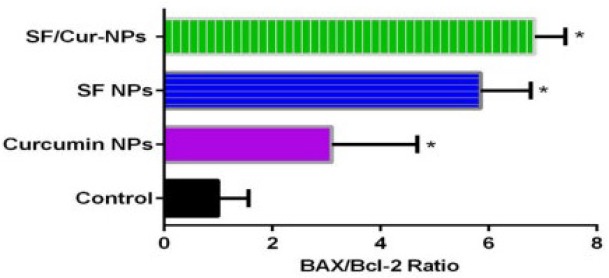
Statistical analysis of BAX/Bcl-2 Ratio results that were treated by SF/CUR-codelivery PEGylated Fe3O4@Au NPs nanoparticles, CUR-NPs, SF-NPs and Fe3O4@Au NPs as control. Mean±SD n=5

*Linearity and LOQ*

The method produced linear responses throughout the SF and CUR concentration range of 0.1-1 mg/mL for SF and CUR which is suitable for intended purposes. A typical linear regression equation of the method was: y = 33153x -0.053, for SF and y = 30771 x +0.0944, for CUR, with x and y representing concentration (mg/mL) and peak height (in arbitrary units), respectively; and the regression coefficient (r) was 0.991.

The lower limit of quantification for SF and CUR were proved to be 0.1 mg/mL. The lower limit of detection for SF and CUR were 0.05 mg/mL.

*Within-run, between run variations, accuracy and recovery*

The within-run precision, between run precision, accuracy and recovery of the developed UV-VIS method as well as the corresponding absolute recoveries are shown in Table 2.

*3.2. Encapsulation efficiency and drug loading of SF and CUR*

The efficiency of encapsulation onto the PEGylated Fe_3_O_4_@Au NPs was determined at 1 and 3 h at pH = 7 as described above. The encapsulation efficiency of SF PEGylated Fe_3_O_4_@Au NPs was 81.20 ± 0.18%, and CUR was 83.72 ± 0.14%, respectively. The loading efficiency of SF and CUR to NPs was 16.74% and 17.32% respectively. Three hours was considered as optimal times for collection the SF and CUR loaded samples. 

*The release profile of SF and CUR*

*In-vitro* SF and CUR release experiments were performed using a dialysis bag method at pH 7.4 and 5.4. The selected pH values indicate the pH of normal blood (pH = 7.4) and a cancer cellʹs environment (pH = 5.4). As controls, the release of free CUR and free SF was studied to verify that the diffusion of drug molecules across the dialysis membrane was not a rate-limiting step during the release process. Free CUR and free SF was observed to be rapidly released and reached its peak of 85.90% and 85.14% respectively of the total in the first 10 h. The results are expressed an SF and CUR release of approximately 3% and 31% in physiologic and 44% and 41% in acidic condition from PEGylated Fe_3_O_4_@Au NPs at the first 12 h which uniformly increases and reaches approximately 66% and 75% for SF and 62% and 68% for CUR after 120 h in normal and acidic conditions, respectively (Figure 5). These results illustrated that the release rate of SF and CUR in an acidic condition was much higher than at a physiologic pH suggesting the SF and CUR loaded MNPs will be more efficient in cancerous cell environments.

*In-vitro cytotoxicity*

First, the cytotoxicity of free SF and CUR on MCF-7 and 4T1 cells was studied. The results are presented in Figure 6. Both CUR and SF could kill cancer cells while the former showed higher efficiency. The IC50s of CUR were 50.68 μg/mL and 58.57 μg/mL on MCF-7 and 4T1 cell lines, respectively; and those for SF were 26.1 μg/mL and 29.5 μg/mL, respectively at 12 h. The IC50s of CUR were 42.78 μg/mL and 46.5 μg/mL on MCF-7 and 4T1 cell lines, respectively and those for SF were 14.1 μg/mL and 17.5 μg/mL, respectively at 24 h. The IC50s of CUR were 37.95 μg/mL and 37.35 μg/mL on MCF-7 and 4T1 cell lines, respectively, and those for SF were 11.1 μg/mL and 14.5 μg/mL, respectively at 48 h. The IC50s of CUR were 35.85 μg/mL and 32.75 μg/mL on MCF-7 and 4T1 cell lines, respectively, and those for SF were 9.8 μg/mL and 12.5 μg/mL, respectively at 72 h .We evaluated the possible synergistic anticancer effects of the free CUR and SF combinations on the MCF-7 cell lines. The anticancer abilities of SF/CUR-encapsulated nanoparticles (the SF/CUR mass ratio was 1:1; the total drug concentration was 60–60 µg/mL) on both the MCF-7 and 4T1 cell lines were studied. The combination treatment indicated obvious synergistic anti proliferation effects on MCF-7 cells (Figure 7).

In order to address the poor water solubility issue of CUR and SF, we used the Fe_3_O_4_@Au nanoparticles to encapsulate them and prepared the SF/CUR-coloaded Fe_3_O_4_@Au nanoparticles. The anticancer abilities of SF /CUR-encapsulated nanoparticles on both the MCF-7 and 4T1 cell lines were then studied. According to our results, the SF/CUR-coloaded Fe_3_O_4_@Au nanoparticles showed anticancer effects in an obvious concentration-dependent manner. Besides, the SF/CUR-co-loaded Fe_3_O_4_@Au nanoparticles had similar anticancer abilities to the free SF/CUR combination. 

*SF/Cur-coloaded Fe*_3_ *O*_4_ *@Au nanoparticles induced apoptosis in human breast cancer cells*

To determine whether the SF/CUR nanoparticle-induced loss of the proliferation capacity and cell viability of human breast cancer cells was associated with the induction of apoptosis, MCF-7 cells were treated with SF nanoparticles (SF concentration: 18 μg/mL), CUR nanoparticles (CUR concentration: 18 μg/mL) and SF/CUR-coloaded Fe_3_O_4_@Au nanoparticles (18 μg/mL for SF and 18 μg/mL for CUR). The numbers of apoptotic cells were evaluated using flow cytometry. As shown in Figure 8, after 48 h of treatment, the early apoptosis rates of the SF nanoparticle- and CUR nanoparticle-treated cells were 40.7% and 36.84%, respectively, while the SF/CUR-loaded Fe_3_O_4_@Au nanoparticle group was 64.7%. The later apoptosis rates region of the SF nanoparticle and CUR nanoparticle-treated cells were 21.9% and 19.8%, respectively, while the SF/Cur-loaded Fe_3_O_4_@Au nanoparticle group was 17.6%. Both the early and later apoptosis data indicated that SF/CUR-loaded Fe_3_O_4_@Au nanoparticles had strong apoptosis-inducing ability. 

*Real-Time PCR and Gene Expression Profile*

To study the effect of SF/CUR-co-loaded Fe_3_O_4_@Au nanoparticles on MCF-7 cell line, the expression of selected genes was analyzed by real-time PCR. The apoptotic pathway is triggered by the release of proteins from the intermediate space of mitochondria; SF/CUR blocks this release by inducing the Bcl-2 family which controls this process including activation of Bax (pro-apoptotic) and inhibition of Bcl-2 (anti-apoptotic) (55, 56). Therefore, we investigated the expression of the target genes in MCF-7 cells after treatment with SF/CUR-coloaded Fe_3_O_4_@Au nanoparticles in compared with PEGylated Fe_3_O_4_@AuNPs, CUR-NPs and SF-NPs. Statistical analysis of real-time PCR clarified that after 72 h of treatment, the SF/CUR-coloaded Fe_3_O_4_@Au nanoparticles significantly decreased the expression of Bcl-2 by 0.28, *P* < 0.01) and Bcl-XL by 0.19, *P *< 0. 01) In compare to the control group. Moreover, SF/CUR-coloaded Fe_3_O_4_@Au nanoparticles considerably (*P* < 0.01) can up-regulated the expression of BAX by 2.705. It also significantly (*P* < 0.01) increased the BAX mRNA levels compared with SF-NPs and CUR-NPs. These data revealed that PEGylated Fe_3_O_4_@Au NPs can improve the SF and CUR effects on up-regulation of BAX expression remarkably. The PEGylated Fe_3_O_4_@Au NP by itself did not vary the expression of Bcl-2, Bcl-XL, or BAX (Figure 9). These results are compatible with our expectation of the SF /CUR behavior and prove that PEGylated Fe_3_O_4_@Au NPs improves the SF and CUR function in controlling apoptosis and metastatic-related gene expression. SF/Cur-coloaded Fe_3_O_4_@Au nanoparticles treatment resulted in an increased expression of Bax, and a decrease in Bcl2 expression with associated with up regulation of the Bax / Bcl2 ratio (Figure 10). In conclusion the results indicate that SF/CUR-coloaded Fe_3_O_4_@Au nanoparticles are an effective therapeutic agent with which can induce apoptosis.

*In-vivo* Toxicity The Fe_3_O_4_@Au nanoparticles, SF/CUR-coloaded Fe_3_O_4_@Au nanoparticles, void CUR and free SF were intravenously injected to female BALB/c mice at a various dose for multiple and single modes for evaluation of possible toxicities of groups. The body weight of mice was not significantly different from control group (injected with PBS) in all groups after two weeks. Also, no mice died during the whole observation period. Histological investigation was performed to evaluate toxicity in kidneys and livers. As the results show in supplementary Figure 2, no certain toxicity, necrosis or immune response activation were obtained in all tissue samples. This demonstrated that prepared Fe_3_O_4_@Au nanoparticles and SF/CUR-coloaded Fe_3_O_4_@Au nanoparticles were safe *in-vivo*.

## Conclusion

In this study, we developed a novel combination of SF and CUR with MNPs as a potential cancer chemo-preventive drug to increase SF and CUR cytotoxic and anticancer effects on the MCF-7 cell line. Besides, the combination of the two agents might improve their therapeutic potentials through synergistic effects (57, 58). In this study, SF and CUR were co encapsulated into Fe_3_O_4_@Au that creating SF/CUR-co-loaded Fe_3_O_4_@Au nanoparticles. To ensure successful synthesis of SF and CUR loaded PEGylated Fe_3_O_4_@Au NPs, the characterization was performed by various techniques, including TEM, SEM, EDX and VSM. Moreover, MTT assay, flow cytometry analysis and real-time PCR were applied to survey the anti-cancer and anti-metastatic effects of SF and CUR loaded PEGylated Fe_3_O_4_@Au NPs. The statistical analyses of the results represent significant increases in amounts of apoptosis, necrosis and cell death in SF and CUR -loaded PEGylated Fe_3_O_4_@Au NPs compared with free SF and CUR or PEGylated Fe_3_O_4_@AuNPs on MCF-7 and 4T1 cells. Furthermore, the designed PEGylated Fe_3_O_4_@Au NPs promote the effects of SF and CUR on converting BAX, Bcl-2 and Bcl-XL gene expression to increase apoptosis. The prepared SF/CUR-coloaded Fe_3_O_4_@AuNPs nano-carriers showed a small size, slow-releasing behavior and stability. Obvious synergistic anticancer effects on MCF-7 and 4T1 breast carcinoma cells were obtained *in-vitro* through co-encapsulation. Our results showed that SF/CUR-coloaded Fe_3_O_4_@AuNPs nanoparticles causes a decrease in cell viability and induces apoptosis by increasing Bax/Bcl-2 ratio.
